# Hyperactivation of β-catenin rewires mitochondrial fuel selection in liver cancer cells

**DOI:** 10.64898/2026.10.18.759986

**Published:** 2026-10-20

**Authors:** Luis Cedeño-Rosario, Queren A. Alcantara, Nicole A. Sierra, Praveena Prasad, Jillian Wright, Geanette Lam, Nimo Abdi, Jaide Florence, Alex J. Bott, Katarina Heyden, Joshua C. Youngs, Álvaro J. Narbona-Pérez, Lorenzo Gastelum Mena, Monika A. Makurath, Jesse N. Velasco-Silva, Mi-Young Jeong, Sarah Fogarty, Gregory S. Ducker, Jennifer Lippincott-Schwartz, Melanie R. McReynolds, Jared Rutter

## Abstract

Metabolic state is tightly linked to cell fate, and expression of the mitochondrial pyruvate carrier (MPC), which imports pyruvate into mitochondria, is a robust marker of it. The transcriptional regulators that set metabolic state, however, remain poorly defined. In the liver, β-catenin is one of the determinants of metabolic zonation, and activating *CTNNB1* mutations are frequent in hepatocellular carcinoma. Here we show that β-catenin regulates metabolic state in liver cancer cells. An unbiased CRISPR screen that uses endogenous *MPC1* transcript abundance as the marker identifies *CTNNB1* . Activating β-catenin pharmacologically or genetically reduces MPC1 and MPC2 transcript and protein levels, and MPC expression is also low in β-catenin-driven liver tumors in vivo. Beyond the MPC, β-catenin activation impairs assembly of respiratory Complexes III and IV, reduces mitochondrial DNA content and respiratory capacity, elongates mitochondrial networks and remodels the lipidome. Stable isotope tracing shows reduced entry of glucose- and lactate-derived carbon into the TCA cycle alongside increased oxidation of palmitate and oleate. These findings identify β-catenin as a regulator of metabolic state that drives a fuel switch from carbohydrate to fatty acid oxidation in liver cancer cells.

